# The kinase LRRK2 is differently expressed in chronic rhinosinusitis with and without nasal polyps

**DOI:** 10.1186/s13601-018-0194-y

**Published:** 2018-03-12

**Authors:** Yue Ma, Chunquan Zheng, Le Shi

**Affiliations:** 1 Department of Otolaryngology-Head and Neck Surgery, Eye Ear Nose and Throat Hospital, Fudan University, 83 Fenyang Road, Xuhui District, Shanghai, 200031 People’s Republic of China; 2 Shanghai Key Clinical Disciplines of Otorhinolaryngology, Shanghai, People’s Republic of China

**Keywords:** LRRK2, NRON, Long non-coding RNA, Chronic rhinosinusitis, Pro-inflammatory cytokine

## Abstract

**Background:**

Chronic rhinosinusitis (CRS), commonly divided into CRS with nasal polyps (CRSwNP) and without nasal polyps (CRSsNP) is an inflammatory disease which mechanism remain unclear. Leucine-rich repeat kinase 2 (LRRK2) has been proved to be a negative regulator of inflammation response while its role in pathogenesis of CRS has yet to be revealed. This research study was designed to investigate the relationship between the expression level and biologic role of LRRK2 in CRS.

**Methods:**

Expression of LRRK2 mRNA and noncoding repressor of NFAT (NRON) were examined by qRT-PCR. Protein levels of LRRK2 were performed by western blot and immunohistochemistry. Nuclear factor of activated T cells (NFAT) nuclear translocation was analyzed by immunohistochemistry. Additionally, LRRK2 mRNA and NRON expression in response to specific inflammatory stimulation was measured in human nasal epithelia cells (HNECs).

**Results:**

The expression of LRRK2 was increased in CRSsNP patients (*p*  <  0.05) and positively correlated with the expression levels of CD3 and Charot-Leyden crystal. Meanwhile, the NRON expression level is much lower in CRSsNP patients compared to both the control group and CRSwNP group (*p*  <  0.05). Marked enhanced NFAT nuclear localization was observed in CRSwNP groups compared with the CRSsNP and control group (*p*  <  0.0001). And the over-expression of LRRK2 was significantly regulated by lipopolysaccharide (LPS) in HNECs (*p*  <  0.05). Moreover, IL-17A can increase LRRK2 expression and suppress NRON expression in vitro and dexamethasone can rescue the NRON inhibition.

**Conclusion:**

LRRK2 and NRON may play different role in CRSsNP and CRSwNP. The molecular mechanisms identified here may aid in the design of novel therapeutic strategies to improve clinical outcomes.

## Background

In general, chronic rhinosinusitis (CRS) is an inflammatory disease that is composed of CRS with nasal polyps (CRSwNP) and without nasal polyps (CRSsNP). Although their pathogenesis is not yet clear, it is commonly acknowledged that the two types of CRS possess distinct inflammation and remodeling patterns [1, 2]. CRSsNP is characterized by Th1-biased inflammation and an elevated expression of transforming growth factor (TGF-β1), while CRSwNP is characterized by a Th2-biased inflammation [3]. Meanwhile, the dysregulation of innate immunity has been regarded as the key point for the initiation and perpetuation of inflammatory responses in CRS patients [4]. The toll-like receptor signaling pathway induced by lipopolysaccharide (LPS)has been reported as one of critical factors in the pathogenesis of CRS, but the mechanism remains unclear. Thus, there is an urgent need for a new biomarker that can further explain this mechanism.

Leucine-rich repeat kinase 2 (LRRK2), also known as Dardarin, is a large complex protein that contains several domains [5–7]. Accumulating evidence has revealed that LRRK2 is involved in regulating inflammatory processes [8–10]. Participation in the signaling of IFN-γ [11, 12], enhancement of NF-kB-dependent transcription, and interference in reactive oxygen species (ROS) production all indicate that LRRK2 may play a pivotal role in the anti-microbial process [11].

It has already been identified that LRRK2 negatively regulates NFAT by interacting with NRON, a known repressor of NFAT. Long non-coding RNA (lncRNA) NRON and 11 additional proteins, including LRRK2, constitute a protein-RNA complex which could directly inhibit the translocation of NFAT to the nucleus [13, 14] such that cell activation and the proceeding immune reactivity are suppressed. For example, enhanced nuclear location of NFAT was associated with more severe colitis in LRRK2-deficient mice [14].

Therefore, LRRK2 is a negative regulator of this inflammation response. These findings implied that the changes in LRRK2 expression levels resulting from extrinsic signals may play a vital role in the regulation of immune responses. However, the role of LRRK2 in the pathogenesis of CRS has yet to be revealed. Our present study was designed to investigate the relationship between the expression level and biologic role of LRRK2 in CRS.

## Methods

### Subjects

The subjects (n = 74) were all recruited from the Department of Otolaryngology-Head and Neck Surgery, Eye, Ear, Nose, and Throat Hospital, Fudan University. All were CRS patients who had not taken oral and/or topical corticosteroids or any other sinonasal medications for at least 1 month prior to the study and were diagnosed based on previously published criteria [15]. NPs were obtained from CRSwNP patients (n = 34), and nasal mucosa of middle turbinates were from CRSsNP patients (n = 23). At the same time, the nasal mucosa of inferior turbinates were collected from those patients (n = 17) who had no clinical symptoms or radiographic evidence of CRS and who underwent a septoturbinoplasty. Patients responding positively to a skin-prick test were diagnosed with an allergy. All of the patients’ clinical data are presented in Table 1. The exclusion criteria for the study group ran as follow: age < 18 or > 80 years, a diagnosis of cystic fibrosis, Churg-Strauss syndrome, immunodeficiency, or autoimmune disease.

**Table 1:** Characteristics of included subjects

	Control subjects	Patients with CRSsNP	Patients with CRSwNP
No. of patients	17	23	34
Sex, male/female	12/5	12/11	20/14
Age (years), mean (SD)	36 (8)	52 (17)	37 (13)
Atopy, no.	0	4	6
Asthma, no.	0	0	2
Aspirin intolerance, no.	0	0	0
Smoking, no.	0	10	16
Operation history, no.	0	0	2
Methodologies used			
Tissue IHC	10	16	25
Tissue mRNA	15	21	30
Tissue western blot	9	9	9

This study obtained permission from the local ethical committee of the Otolaryngology-Head and Neck Surgery, Eye, Ear, Nose, and Throat Hospital, Fudan University, and informed consent was signed by every subject.

### RNA extraction and real-time polymerase chain reaction

Total RNA was extracted using TRIzol reagent (Invitrogen) according to the manufacturer’s instructions. Then, PrimeScript RT master mix (Takara) was used to synthesize complementary DNA (cDNA). An ABI 7900 Sequence Detection System (ABI) was used to perform qRT-PCR with SYBR Green chemistry. Table 2 showed the primers (Sangon Biotech). The expression of each gene was calculated using the comparative threshold cycle (2^−ΔΔCT^) method.

**Table 2:** Primers

Name	Sense	Antisense
GAPDH	CAAGGTCATCCATGACAACTTTG	GTCCACCACCCTGTTGCTGTAG
LRRK2	GGATGTTGGTGATGGAGTT	GGCTGAGTGGAGGTATCT
NRON	AACAACCCAGCAAGGGAAGTAG	AAGAGCATGAACGCACATCCTAG
CD3	TAGAGGAACTTGAGGACAGA	GCAGAGTGGCAATGACAT
Tryptase	TCTGAAGCAGGTGAAGGT	AGTCCAAGTAGTAGGTGACA
CLC	TTGTCTACTGGTTCTACTGT	CAATGTCTGATTCCTCCTTC
CXCR1	ATGCTGTTCTGCTATGGATT	CGATGAAGGCGTAGATGAT
CD68	CTCCAGCAGAAGGTTGTC	TGATGAGAGGCAGCAAGA

### Immunohistochemistry (IHC) staining

Immunohistochemistry was performed following the protocol for the streptavidin-biotin complex (SABC) kit (Weiao Biological Technology). The sections were incubated with primary antibody (polyclonal rabbit anti-human LRRK2; Abcam; 1:200 dilution; polyclonal goat anti-human NFAT1; Abcam; 1:100 dilution) at 4 °C overnight. 3′3-Diaminobenzidine (DAB) was used for the final visualization. To analyze LRRK2 expression, two pathologists scored the results independently according to the immunostaining intensity scale, which ran as follows: 0 = absent; 1 = mild; 2 = moderate; and 3 = marked. The numbers of immuno-positive cells within the samples were also counted in at least five random areas at a 400× magnification, and at the same time, the percentage of immuno-positive cells was scored according to the standard scale as follows: 0 (0–9%); 1 (10–25%); 2 (26–50%); 3 (51–75%); and 4 (> 76%). Multiplication of the two abovementioned scores provided the final score for each sample. The highest final score was 12 while the lowest was 0. To analyze NFAT nuclear translocation, the percentage of cells with NFAT1 nuclear staining in all immuno-positive cells were also counted in five random areas at a 400x magnification.

### Western blots

Protein samples (30 μg) were separated by electrophoresis using 10% sodium dodecyl sulfate polyacrylamide gels and then transferred to polyvinylidene difluoride (PVDF) membranes for incubation with anti-LRRK2 (Abcam; 1:1000) antibody. Image J (NIH) analysis and processing software was used to quantify data, which was expressed as densitometry units (DU). The expression of *β*-actin (Abcam; 1:1000) was regarded as an internal reference.

### Cell culture and stimulation

Following a previously established protocol, HNECs (Human Nasal Epithelia Cells) isolated from the middle turbinates of CRSsNP patients were cultured [16]. Briefly, nasal specimens were immersed in DMEM/F12 media (Hyclone) containing 1.4 mg/ml protease K and 0.1 mg/ml DNase for a 1.5 h incubation at 37 °C. Next, all cells were collected and immersed in DMEM/F12 (Hyclone) containing 1% ITS for 2 h at 37 °C before being cultured in BEGM medium (Lonza).

When 80–90% confluence was reached, fresh media without hydrocortisone was added in the presence of the following stimulators or control PBS for 12 h: the recombinant cytokines human IFN-γ (100 ng/mL), IL-4 (100 ng/mL), IL-13 (100 ng/mL), IL-17A (100 ng/mL), TGF-β (10 ng/mL), and IL-1α (100 ng/mL; all purchased from Peprotech); the TLR agonists LPS (500 ng/mL, from *Escherichia coli* serotype 0111: B4; purchased from Sigma) and the glucocorticoid dexamethasone (Sigma; 10 μg/mL). After stimulation, HNECs were collected for qRT-PCR analysis.

### Statistical analysis

Statistical analyses were performed by SPSS v22.0 software (IBM Corporation). The data were presented as medians and interquartile ranges. Tests for Gaussian distribution were performed by Kolmogorov–Smirnov test. Differences between groups were evaluated by one-way analysis of variance and either by the two independent sample t test or the Mann–Whitney U test. The correlation analysis was performed to assess the correlation between two groups by Spearman’s rank correlation. *p* < 0.05 was regarded as statistically significant.

## Results

### NRON and LRRK2 mRNA levels in nasal tissues

**Fig. 1:**
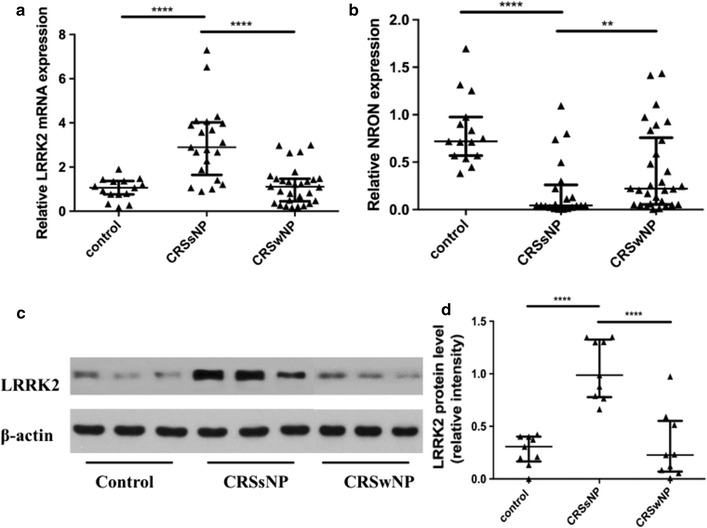
The expression of LRRK2 and NRON in nasal tissues. **a** LRRK2 mRNA levels was upregulation in CRSsNP groups compared with CRSwNP groups and the control group; **b** NRON expression was significantly reduced in CRSsNP patients; **c** representative western blot results of LRRK2; **d** densitometric analysis. **p < 0.01, ****p < 0.0001

As showed in Fig. 1a, significant upregulation of LRRK2 mRNA levels was found in CRSsNP groups but not in CRSwNP groups compared with the control group (*p* < 0.0001), and NRON levels were significantly higher in the inferior turbinate than in the middle turbinate of CRSsNP groups (*p* < 0.0001) and in NPs (*p* < 0.01) (Fig. 1b).

### LRRK2 protein levels in nasal tissues

To verify the results at the protein level, western blots were performed. Strong bands for LRRK2 were observed in CRSsNP groups, whereas weak bands were found in the CRSwNP group and in the control group (Fig. 1c). The LRRK2 protein level was significantly higher in the CRSsNP group than in the CRSwNP group and the control group (Fig. 1d; *p* < 0.0001).

### LRRK2 and NFAT immunoreactivity in nasal tissues

**Fig. 2:**
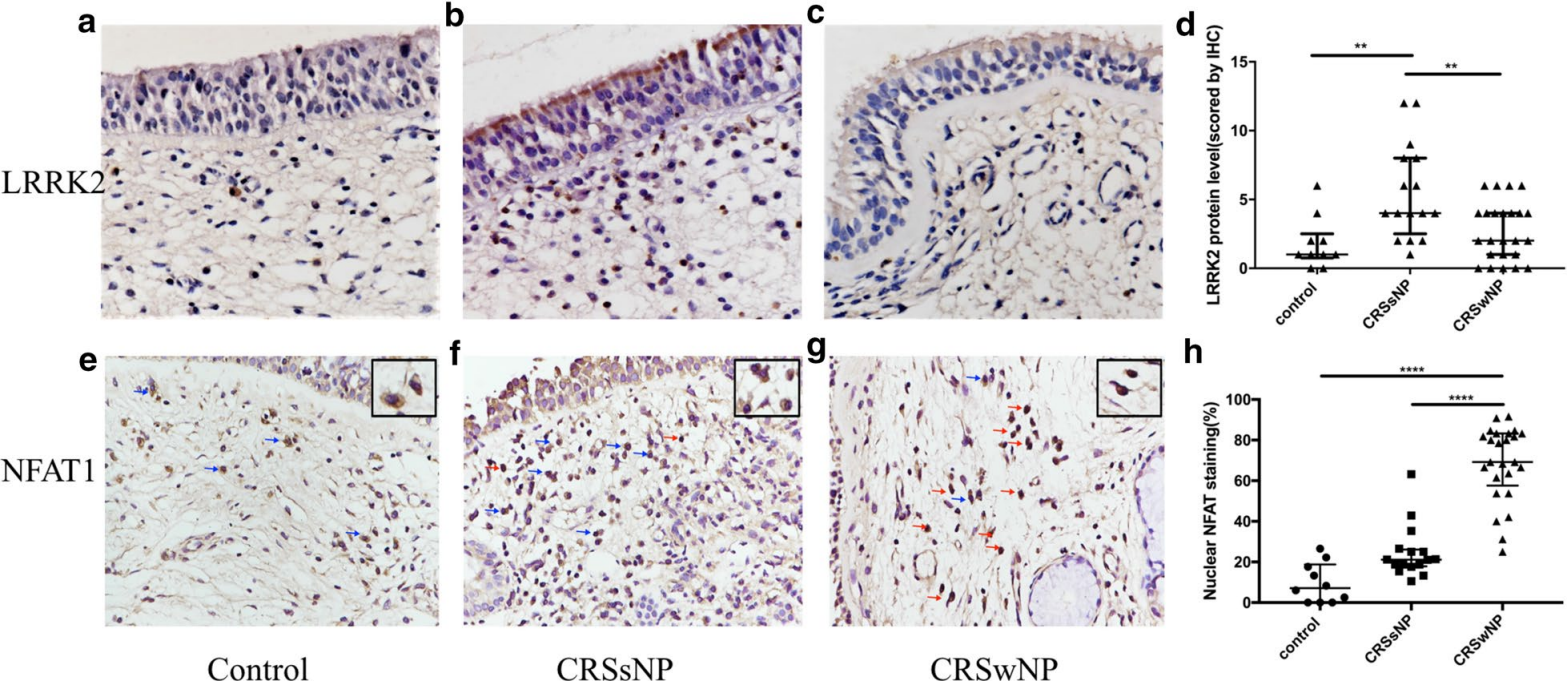
LRRK2 inhibits NFAT1 nuclear translocation. (**a**–**c**; ×400 magnification) LRRK2 expression and distribution in control, CRSsNP and CRSwNP groups; **d** LRRK2 was significantly overexpressed in the CRSsNP group compared with the control group and CRSwNP group; (**e**–**g**; ×400 magnification) NFAT1 localization in control, CRSsNP and CRSwNP groups; blue arrows indicate cells with cytosolic NFAT1 staining, red arrows indicate cells with nuclear NFAT1 staining; the top right corner is an enlargement of immuno-positive cells; **h** the percentage of cells with NFAT1 nuclear staining was much higher in CRSwNP group compared with the control group and CRSsNP group. **p < 0.01, ****p < 0.0001

Immunohistochemistry was performed to further confirm the results pertaining to the protein levels of LRRK2 in the various samples. As depicted by immunohistochemistry staining (Fig. 2a–c), LRRK2 was significantly overexpressed in the CRSsNP group compared with the control group and CRSwNP group. The cytoplasmic or nuclear staining of LRRK2 was mainly located at the nasal epithelium and in submucosal inflammatory cells. In comparison with control groups and NPs, quantitative analysis of LRRK2 revealed an obvious elevation in immuno-labeling of LRRK2 in the CRSsNP group (Fig. 2d; *p *< 0.01). NFAT nuclear translocation was also detected by immunohistochemistry (Fig. 2e–g). Marked enhanced NFAT nuclear localization was observed in CRSwNP groups compared with the CRSsNP group and control group (Fig. 2h; *p *< 0.0001).

### Detection of LRRK2 producing cells in nasal mucosa

**Fig. 3:**
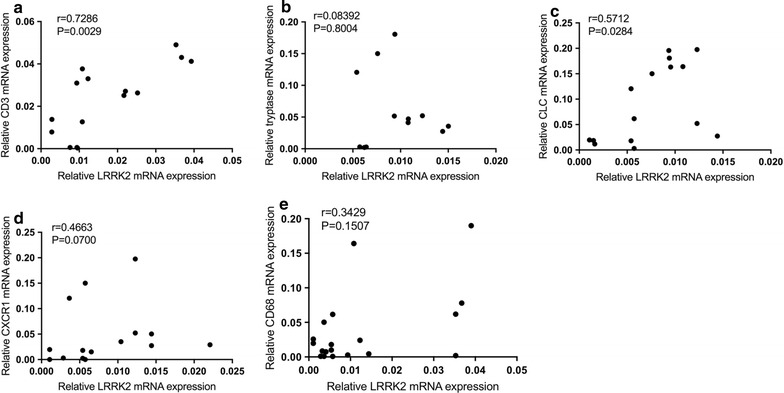
Correlation of LRRK2 with cell specific markers in nasal mucosa of CRSsNP patients (**a**–**e**); the expression of LRRK2 and cell specific markers was analyzed by real-time PCR. The correlations were assessed by using the Spearman rank correlation

These results revealed that LRRK2+ cells were highly accumulated in the submucosal region of CRSsNP tissues and that the mRNA and protein expression levels of LRRK2 were also significantly higher in CRSsNP tissues (Fig. 2). Next, the expression of LRRK2 and the markers of inflammatory cells in CRSsNP were assessed by qRT-PCR and the relationship between these markers were analyzed by Spearman’s rank correlation. The results demonstrated that the mRNA expression levels of LRRK2 in CRSsNP tissue were significantly and positively related to the expression of CD3 (r = 0.7286; *p* = 0.0029), Charot–Leyden crystal (CLC; r = 0.5712; *p* = 0.0284), CD68 (r = 0.146; *p* < 0.05), but not CXCR1, tryptase and CD68, which indicated that the expression of LRRK2 may derive from T cells, eosinophils (Fig. 3).

### LPS and pro-inflammatory cytokines differentially regulate LRRK2 and NRON expression in human nasal epithelial cells (HNECs) in vitro

**Fig. 4:**
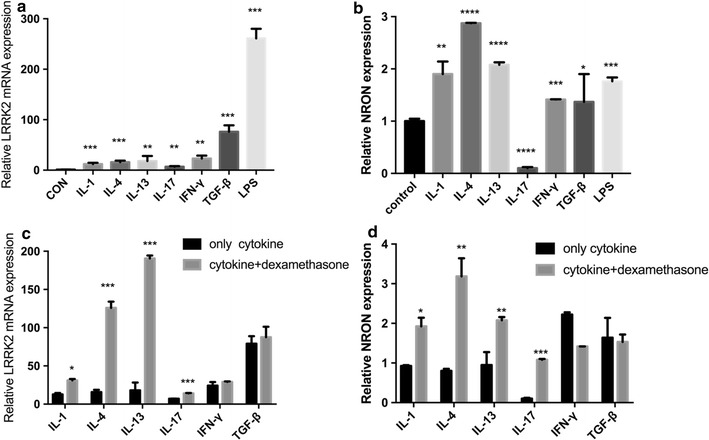
LRRK2 mRNA and NRON expression in cultured HNECs in response to pro-inflammatory cytokines, LPS and dexamethasone. **a** LRRK2 mRNA, **b** NORN expression after a 12 h stimulation; **c** LRRK2 mRNA, **d** NORN expression after pro-inflammatory cytokines stimulation with or without dexamethasone. Results represent mean values from 3 independent experiments. Data are expressed as means (SEMs). *p < 0.05, **p < 0.01, ***p < 0.001, ****p < 0.0001

To further expose the role and mechanism of the LRRK2 signaling pathway in the nasal mucosa of CRS, the effects of TLR activation and pro-inflammatory cytokine stimulation were examined in cultured human nasal epithelial cells. As presented in Fig. 4a, mRNA expression levels of LRRK2 were significantly increased after stimulating the HNECs with LPS, the TLR agonists (*p* < 0.001). Meanwhile, stimulation with IL-17A increased very low LRRK2 mRNA expression (*p* < 0.01) compared with stimulation by the other pro-inflammatory cytokines, including IFN-γ, IL-4, IL-13, IL-1α, and TGF-β which also increased LRRK2 expression (*p* < 0.01). In contrast, NRON levels were significantly inhibited by stimulation with IL-17A (*p* < 0.001; Fig. 4b).

### Glucocorticoid treatment increases LRRK2 expression in vitro

Because glucocorticoid treatment is recommended as one of the primary treatment of choice for patients with CRS, the effect of glucocorticoids on LRRK2 mRNA and NRON expression in HNECs was examined. After incubating cells with dexamethasone, LRRK2 mRNA and NRON expression was more upregulated in the presence of pro-inflammatory cytokines (IL-17A, IL-4, IL-13, IL-1α; *p* < 0.05) than without dexamethasone stimulation (Fig. 4c, d). In contrast, NRON expression was lower when the cells were stimulated with IFN-γ plus dexamethasone compared to only IFN-γ stimulation.

## Discussion

This study demonstrated that the expression of LRRK2 was increased in patients suffering from CRSsNP (Figs. 1, 2), and the over-expression of LRRK2 was regulated by LPS in HNECs (Fig. 4). Moreover, LRRK2 expression in CRSsNP tissues was correlated with the expression levels of CD3, CLC, and CD68, which suggested that T cells, eosinophils, and macrophages may be the main LRRK2-producing cells in nasal mucosa (Fig. 3). Meanwhile, NRON expression level is much lower in CRSsNP patients compared to both the control group and CRSwNP group (Fig. 1). Its expression can be strongly suppressed by stimulation with IL-17A in vitro, and dexamethasone can rescue this phenomenon.

CRS, commonly divided into CRSwNP and CRSsNP, is characterized by chronic inflammation of the paranasal sinuses. Due to the poor awareness of the initiation and progression of CRS, the development of effective treatments for this disease remains stagnant [17]. LRRK2 can be physically associated with NRON and this protein-RNA complex has been proven to be a NFAT suppressor in many inflammatory diseases such as inflammatory bowel disease [18], and LRRK2 deficiency can lead to a more serious inflammation response. In the results presented herein, considerable data indicates that the up-regulation of LRRK2 expression in CRSsNP is obviously higher than in the CRSwNP group, and the percentage of cells with NFAT nuclear staining in CRSsNP is much lower in CRSwNP group (Fig. 2), which suggests that a lower expression of LRRK2 exhibits less of an inhibitory effect on NFAT. Moreover, this data supports that the idea that the defection of innate immunity may lead to a robust adaptive immune response, which triggers the generation of pro-inflammatory cytokines and the development of chronic inflammation [19].

NRON is a cytoplasmic lncRNA which has previously been reported to be highly expressed in human immune cells including macrophages, dendritic cells, and neutrophils along with its vital role in T cell cytokine production. NRON acts as a scaffold to maintain the structure of a ribonucleoprotein complex which can retain the transcription factor NFAT in the cytoplasm [13], meaning that NRON plays a significant role in the immune response. However, in this study, the expression level of NRON is lower in CRSsNP group, which indicates that in the RNA-protein complex, LRRK2 may play a much more important role in the generation and the development of CRS than NRON.

Within the immune system, the expression of LRRK2 has been reported in lymphocytes, dendritic cells, and macrophages [11]. In this study, LRRK2-producing cells were identified in CRSsNP nasal mucosa. Considerable data had already shown that T cells and eosinophils may be the main LRRK2-producing cells in CRSsNP nasal mucosa, which was in line with the published reports.

It has also been reported that the induction of LRRK2 expression could be triggered in mouse bone marrow-derived macrophages under the stimulation of toll-like receptor 4 [20], whereas opposite findings were issued in another study [14]. Hence, to further explore the mechanism of LRRK2 and NRON expression in CRS, HNECs were incubated with LPS and pro-inflammatory cytokines (IFN-γ, TGF-β, IL-1α, IL-4, IL-13, IL-17A). The results showed that IL-17A can increase LRRK2 and suppress NRON expression which were coincidence with the expression of LRRK2 and NRON in nasal mucosa of CRSsNP patients, while other cytokines can induce both LRRK2 and NRON. The results indicate that IL-17A may play a significant role in the LRRK2 signaling pathway in CRSsNP patients, while both Th1 and Th2 cytokines participate in the inflammation activities of CRSsNP nasal mucosa. Further studies are urgently needed to confirm this theory.

Notably, this study found that dexamethasone universally enhanced the induced role of pro-inflammatory cytokines (IL-13, IL-1α, IL-4) in LRRK2 mRNA and NRON expression. What’s more, it can rescue IL-17A cytokine-induced NRON suppression. Although the underlying molecular mechanisms require further characterization, this finding demonstrates that glucocorticoid treatment can make more LRRK2-NRON complex so that it can play a more significant role in the inhibition of NFAT translocation. Then, the inflammation response will be inhibited.

## Conclusions

In summary, it has been revealed here that LRRK2 is elevated in patients with CRSsNP, while NRON is lower in this group. LRRK2 can strongly inhibit the nuclear function of NFAT. IL-17A may play a significant role in the LRRK2 signaling pathway in CRSsNP patients. Further investigation will concentrate on the interaction of LRRK2 and NRON in CRS to reveal their effects on inflammation and the immune system. The molecular mechanisms identified here will help clarify the pathogenic processes involved in these two CRS subsets, as well as aid in the design of novel therapeutic strategies to improve clinical outcomes.

## Abbreviations

CRS: chronic rhinosinusitis
LPS: lipopolysaccharide
LRRK2: leucine-rich repeat kinase 2
NRON: noncoding repressor of NFAT
NFAT: nuclear factor of activated T cells
ROS: reactive oxygen species
lncRNA: long non-coding RNA
IHC: immunohistochemistry
SABC: streptavidin-biotin complex
PVDF: polyvinylidene difluoride
DU: densitometry units
HNECs: human nasal epithelia cells
CLC: Charot–Leyden crystal
